# Transcription factor DREF regulates expression of the microRNA gene bantam in Drosophila melanogaster

**DOI:** 10.18699/vjgb-24-20

**Published:** 2024-04

**Authors:** M.B. Schwartz, M.M. Prudnikova, O.V. Andreenkov, E.I. Volkova, I.F. Zhimulev, O.V. Antonenko, S.A. Demakov

**Affiliations:** Institute of Molecular and Cellular Biology of the Siberian Branch of the Russian Academy of Sciences, Novosibirsk, Russia; Institute of Molecular and Cellular Biology of the Siberian Branch of the Russian Academy of Sciences, Novosibirsk, Russia; Institute of Molecular and Cellular Biology of the Siberian Branch of the Russian Academy of Sciences, Novosibirsk, Russia; Institute of Molecular and Cellular Biology of the Siberian Branch of the Russian Academy of Sciences, Novosibirsk, Russia; Institute of Molecular and Cellular Biology of the Siberian Branch of the Russian Academy of Sciences, Novosibirsk, Russia; Institute of Molecular and Cellular Biology of the Siberian Branch of the Russian Academy of Sciences, Novosibirsk, Russia; Institute of Molecular and Cellular Biology of the Siberian Branch of the Russian Academy of Sciences, Novosibirsk, Russia

**Keywords:** microRNA, genetic regulation, mutagenesis, transcription, transcription factors, микроРНК, генетическая регуляция, мутагенез, транскрипция, факторы транскрипции

## Abstract

The bantam gene encodes a vital microRNA and has a complex expression pattern in various tissues at different stages of Drosophila development. This microRNA is involved in the control of normal development of the ocular and wing imaginal discs, the central nervous system, and also in maintaining the undifferentiated state of stem cells in the ovaries of adult females. At the cellular level, bantam stimulates cell proliferation and prevents apoptosis. The bantam gene is a target of several conserved signaling cascades, in particular, Hippo. At the moment, at least ten proteins are known to directly regulate the expression of this gene in different tissues of Drosophila. In this study, we found that the bantam regulatory region contains motifs characteristic of binding sites for DREF, a transcription factor that regulates the expression of Hippo cascade genes. Using transgenic lines containing a full-length bantam lethality-rescuing deletion fragment and a fragment with a disrupted DREF binding site, we show that these motifs are functionally significant because their disruption at the bantam locus reduces expression levels in the larvae and ovaries of homozygous flies, which correlates with reduced vitality and fertility. The effect of DREF binding to the promoter region of the bantam gene on its expression level suggests an additional level of complexity in the regulation of expression of this microRNA. A decrease in the number of eggs laid and a shortening of the reproductive period in females when the DREF binding site in the regulatory region of the bantam gene is disrupted suggests that, through bantam, DREF is also involved in the regulation of Drosophila oogenesis.

## Abstract (ru)

Ген bantam кодирует жизненно важную микроРНК и имеет сложный паттерн экспрессии в различных тканях на разных стадиях развития дрозофилы. Эта микроРНК обеспечивает нормальное развитие глазных и крыловых имагинальных дисков, центральной нервной системы, а также участвует в поддержании недифференцированного состояния стволовых клеток в яичниках взрослых самок. На клеточном уровне bantam стимулирует пролиферацию клеток и препятствует апоптозу. Ген bantam является мишенью нескольких консервативных сигнальных каскадов, в частности Hippo. На сегодняшний день известно не менее 10 белков, напрямую регулирующих экспрессию этого гена в разных тканях дрозофилы. В настоящей работе мы обнаружили, что регуляторная область bantam содержит мотивы, характерные для сайтов связывания DREF – транскрипционного фактора, который регулирует экспрессию генов каскада Hippo. Используя трансгенные линии, содержащие полноразмерный фрагмент, спасающий летальность делеции bantam, и фрагмент с нарушенным сайтом связывания DREF, мы показали, что эти мотивы имеют функциональное значение, поскольку их нарушение в локусе bantam снижает уровень экспрессии в личинках и яичниках гомозиготных мух, что коррелирует со сниженной жизнеспособностью и фертильностью. Влияние связывания DREF с промоторной областью гена bantam на уровень его экспрессии предполагает дополнительный уровень сложности регуляции экспрессии этой микроРНК. Снижение количества откладываемых яиц и сокращение репродуктивного периода у самок при нарушении сайта связывания DREF в регуляторной области гена bantam позволяют предполагать, что через bantam DREF также участвует в регуляции оогенеза дрозофилы.

## Introduction

The bantam gene encodes a vital microRNA that is expressed in many tissues throughout the Drosophila life cycle. At the cellular level, bantam stimulates cell proliferation and prevents apoptosis (Brennecke et al., 2003). This microRNA provides normal development of the ocular and wing imaginal discs, the central nervous system, and it is also involved in maintaining the undifferentiated state of stem cells in the ovaries of adult females (Shcherbata et al., 2007; Peng et al., 2009; Reddy, Irvine, 2011; Slattery et al., 2013; Weng, Cohen, 2015).

Expression of the bantam gene is controlled by a wide range of transcription factors that form different assemblies in different tissues at different stages of development. Such conserved morphogens as Notch, Wingless, and Dpp are involved in the regulation of the bantam gene expression in different tissues (Herranz et al., 2008; Oh et al., 2010; Ku, Sun, 2017). Currently, about ten proteins that directly regulate bantam expression in different tissues are known. Dysregulation of bantam leads to abnormal development of many imaginal organs, and problems with vitality and fertility (Hipfner et al., 2002; Brennecke et al., 2003; Shcherbata et al., 2007).

One of the key regulators of bantam expression is the transcriptional coactivator Yorkie (Yki), which provides the necessary level of bantam expression by binding to tissuespecific transcription factors (Peng et al., 2009; Slattery et al., 2013; Nagata et al., 2022). Yki is part of the highly conserved Hippo signaling cascade (Oh et al., 2010). In Drosophila, this cascade suppresses the process of cell division, inducing cell cycle arrest and apoptosis. The cascade begins with the kinase Hippo (Hpo), which triggers the sequential phosphorylation of a number of proteins and, finally, leads to the inactivation of Yki (Huang et al., 2005; Reddy, Irvine, 2011). The hpo gene promoter has been shown to contain DRE motifs (DREF responding element). The promoter of the warts (wts) gene, the second key kinase of the Hippo cascade, also contains DRE motifs. The transcription factor DREF binds to these motifs, enhancing the expression of hpo and wts in the ocular imaginal discs (Fujiwara et al., 2012; Vo et al., 2014). Thus, DREF positively regulates the Hippo cascade and, accordingly, reduces the expression level of bantam.

In our work, we found that the bantam regulatory region contains motifs characteristic of DREF binding sites and tested the consequences of their disruption at the organism level. We examined how disruption of DREF binding sites at the bantam locus affected fly viability and fertility.

## Materials and methods

Obtaining a mutation of the DREF binding site. To obtain the mutation, we used a DNA fragment from the bantam locus of 4709 bp in length (3L:637635-642343, release=r6.23), including DRE and DRE-like motifs. The DRE and DRE-like motifs were isolated from each other using the unique EcoRV restriction site located between them. To mutate each of these motifs, restriction with endonuclease ClaI (AT|CG|AT) was performed, and then the 5ʹ-overhangs were end-filled using a Klenow fragment. This involved the insertion of two nucleotides (CG) into the key part of each motif. Then both parts of the 4709 bp DNA fragment with mutated DRE and DRE-like motifs were ligated and the resulting DNA fragment DREF was inserted at the KpnI and NotI restriction sites into the previously obtained pUni-mod vector containing the attB recombination site (Andreenkov et al., 2016). To obtain the transgenic line “DREF” we used the attP/attB system for specific integration, and a line containing an attP site in the region 10A1-2 of the X chromosome (Andreenkov et al., 2016).

Fly stocks. The “4.7” transgenic line’s genotype was y1, Df(1)w67c23, 10А1-2-“4.7”; banΔ1/ТМ6В. It contained a transgene insertion with a full-length DNA fragment from the bantam locus with a length of 4709 bp into the 10A1-2 region (Schwartz et al., 2019).

The transgenic line “DREF” had the following genotype: y1, Df(1)w67c23, 10А1-2-“DREs_mut”; banΔ1/ТМ6В, and it contained the insertion of a transgene with a modified fragment 4.7 in the 10A1-2 region. The modification of the 4.7 fragment consisted in mutations in potential binding sites for the DREF protein. In all experiments performed, homozygotes for transgene insertion were studied.

The following lines were used as control: “yw” with the genotype y1 Df(1)w67c23; “ban+” with the genotype y1, Df(1) w67c23; +/ТМ6В, where ТМ6В is a balancer chromosome with Tb phenotype – short body of larvae and adults; “Δban” with the genotype y1, Df(1)w67c23 ; banΔ1/ТМ6В.

Lines of flies were kept at +23 °С on standard food with addition of dry yeast

Immunostaining. Indirect immunofluorescence staining of polytene chromosomes was performed according to the previously described protocol (Kolesnikova et al., 2013) using mouse monoclonal antibodies to the DREF protein (kindly provided by C.M. Hart, USA), diluted in a ratio of 1:200, with subsequent coloring by goat-anti-mouse-Alexa 488 antibodies (Thermo Fisher Scientific, # A28175), diluted in a ratio of 1:600.

Fly viability assessment. To determine the viability of transgenic line flies, 5 females and 5 males of the same line were placed in one glass vial. Once every 5 days, the flies were transferred to fresh food, and the experiment continued for a month. Flies of the “TM6” line were used as a control. Based on the results of 3 repetitions during the experiment, each line had on average the following number of descendants: “4.7” – 454 ± 92; “DREF”– 287 ± 112; “ТМ6” – 756 ± 289.

The viability of homozygotes for the banΔ1 deletion in transgenic lines was determined as the ratio of the number of flies with a body of normal length (Tb+) to the total number of hatched offspring. Since the Tb phenotype is identified at the stages of larvae, pupae and adults, it is possible to distinguish flies lacking the balancer, homozygous for the banΔ1 deletion, from heterozygous flies at different stages of development. The “ban+” line, without a transgene and without a deletion, was used as a control. The viability of flies at different developmental stages in transgenic lines was compared with the viability of control flies using Student’s t test, with a preliminary check for normal distribution using the Shapiro–Wilk test.

Female fertility assessment. To determine the fertility of females of transgenic lines, they were crossed with males of the “yw” line. Females of the “yw” line were used as control. In each cross we used 5 females and 5 males. Every day the flies were transferred to fresh food and the number of eggs laid was counted. The experiment continued until the death of the last transgenic female in a vial. The number of eggs during the entire experiment was normalized to the number of females. The experiment was repeated three times. When plotting the fertility dynamics curve, the number of eggs laid by females each day was normalized to the current number of living females. Differences in fertility levels between lines were assessed using Student’s t test, as well as the χ2 test.

Determination of the expression level of mature microRNA bantam. The expression level of mature microRNA bantam was determined by quantitative PCR combined with a reverse transcription reaction (qRT-PCR), adapted for the study of microRNAs through the use of an extended stemloop primer (Chen et al., 2005; Kramer, 2011). We used U6 snRNA as a reference gene (Zhang et al., 2017). To obtain cDNA, we used 5 μg of total RNA, M-MuLV-PH revertase, and accompanying reagents according to the manufacturer’s instructions (Biolabmix). Relative bantam gene expression was determined using the ΔΔСt method. qRT-PCR was carried out using a thermal cycler BioRad C-1000 (USA).

The experiment was done in two biological replicates. We used 30 μl of the following reaction mixtures: for bantam detection – 3 μl of 5 μM ban-F and ban-R primers, 3 μl of 2.5 μM TaqMan-ban probe, 3 μl of 10xAS buffer, 3 μl of 4 μM dNTP, 1 u. a. of Taq polymerase; for U6 detection – 3 μl of 10 μM U6-F and U6-R primers, 3 μl of 2.5 μM TaqMan U6 probe, 3 μl of 10xAS buffer, 3 μl of 4 μM dNTP, 1 u. a. of Taq polymerase, 3 μl of 10 mМ MgCl2 to the final concentration of Mg2+ equal to 2.5 mМ for a reaction. The nucleotide sequences of the primers and probes used in the experiments are given in 5ʹ→3ʹ orientation: ban-SL – gtcgtatccagtgcagggtccgaggtattcgcactg gatacgacaatcag, ban-F – cgccgggcatgagatcattttg, ban-R – cagt gcagggtccgaggt, TaqMan-ban – cgcactggatacgacaatcagcttt, U6-SL – gtcgtatccagtgcagggtccgaggtattcgcactggatacgacggc catgc, U6-F – gccgcatacagagaagatta, U6-R – agtgcagggtc cgaggta, TaqMan-U6 – ttcgcactggatacgacggccatgc.

## Results

The bantam regulatory region contains DREF binding sites

**Fig. 1.**
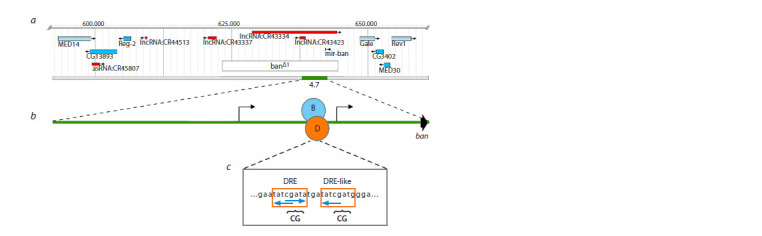
Molecular and genetic organization of the bantam locus. a – DNA fragment 4.7 containing the bantam gene (green rectangle), banΔ1 deletion (white rectangle). The location of other genes in the region (blue and red rectangles); b – the 4.7 DNA fragment organization scheme. Binding sites for DREF and BEAF-32 proteins (ovals D and B, respectively). Curved arrows correspond to the positions of putative promoters of the bantam gene (Brennecke et al., 2003; Qian et al., 2011). The bantam hairpin position (black arrow); c – nucleotide sequence with binding sites for DREF and BEAF-32 proteins. Motifs characteristic of the DREF (orange rectangles) and BEAF-32 (blue arrows) binding sites. Double nucleotide insertions that disrupt DREF binding sites are indicated by curly brackets.

To study the role of DREF in the regulation of bantam gene expression, we used transgenic fly lines. The “4.7” fly line contained a transgene insertion with a 4709 bp long DNA fragment from the bantam locus (Fig. 1, a) into the 10A1-2 region of the X chromosome. This fragment, hereafter referred to as fragment 4.7 (see Fig. 1, b), contains a sequence encoding the bantam miRNA hairpin as well as two putative promoters of the bantam gene (Brennecke et al., 2003; Qian et al., 2011). It has been shown previously that the 4.7 fragment rescues the lethal banΔ1 deletion, removing approximately 21 kb from the bantam locus (Schwartz et al., 2019). In the fragment 4.7, 1.2 kb upstream of the bantam hairpin, we found TATCGATA and TATCGATG motifs corresponding to DRE and DRElike elements, respectively (Ohler et al., 2002). Both motifs are characteristic of binding sites for the transcription factor DREF (see Fig. 1, с).

**Fig. 2.**
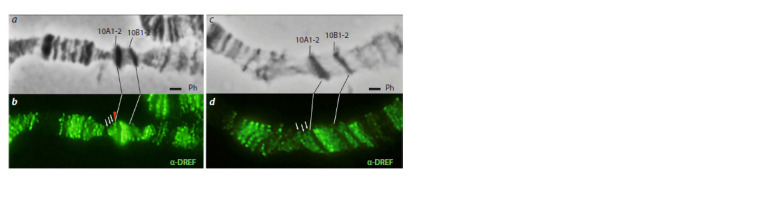
Immunolocalization of the DREF protein in the 10A1-2 region of the X chromosome transgenic fly lines. Fly lines: “4.7” (a, b) and “DREF” (c, d). Microphotographs of polytene X chromosomes of larval salivary glands in phase contrast (Ph) mode and after staining with antibodies against DREF (green). White arrows indicate endogenous DREF localization signals in the 9F region. The red triangle marks an additional localization signal of DREF in the distal part of the 10A1-2 band, corresponding to the localization of the transgene in the “4.7” line. There is no additional signal in the “DREF” line (d ). Scale bar 1 μm.

The “DREF” fly line contained a transgene insertion with the 4.7 fragment with disrupted DREF binding sites. To disrupt the DREF binding sites, we introduced mutations in the DRE (DREF-responding element) and DRE-like motifs (see Fig. 1, c). It should be noted that DRE and DRE-like include CGATA motifs that form the binding site for the insulator protein BEAF-32. The mutations we introduced did not disrupt the CGATA motifs and, accordingly, did not destroy the BEAF-32 binding site. Immunolocalization on polytene chromosomes of Drosophila larvae showed that in line “4.7” in the distal part of the 10A1-2 band at the site of transposon insertion there was an additional DREF localization signal (Fig. 2, b, red arrow). At the same time, this additional signal was absent in the “DREF” line (see Fig. 2, d). It can be assumed that the DRE and DRE-like motifs we found correspond to the binding site of the DREF protein, and the mutations of these motifs we introduced lead to disruption of DREF protein binding.

Disruption of the DREF binding site in the bantam regulatory region affects fly viability

**Fig. 3.**
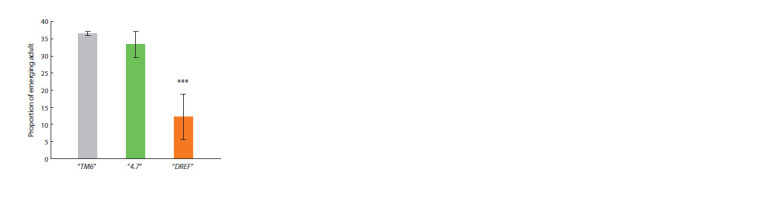
Effect of DREF binding to the bantam regulatory region on adult fly viability in transgenic fly lines. The proportion of adults homozygous for the banΔ1 deletion in transgenic fly lines “4.7” (green column) and “DREF” (orange column). The proportion of adults in the control fly line “TM6” (gray column), containing only native wildtype bantam locus. *** р <0.001.

We found that flies of the “DREF” line survived against the background of the banΔ1 deletion, but the viability of such flies was significantly reduced compared to flies of the “4.7” line (Fig. 3), as well as compared to control “TM6” flies, containing neither the transgene nor the banΔ1 deletion. Moreover, the death of flies homozygous for banΔ1 in the “DREF” line mainly occurred at the late pupal stage, which coincides with the characteristic lethality of the banΔ1 deletion (Brennecke et al., 2003). The use of control containing TM6B balancer allowed taking into account the influence of the balancer itself on the fly viability

**Fig. 4.**
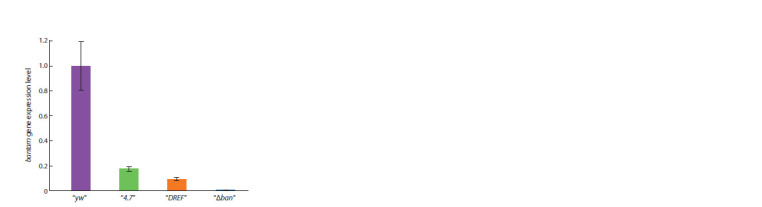
The expression of bantam microRNA in the 3rd instar larvae The amount of mature microRNA bantam in the transgenic lines “4.7” and “DREF” and the control line “Δban”, homozygous for the banΔ1 deletion. The expression of bantam in the control line “yw” was conditionally taken as one. The bantam expression levels were normalized to the expression level of the U6 snRNA reference gene.

In order to determine whether the decrease in viability of transgenic “DREF” flies was associated with bantam gene expression, we examined the expression level of the mature bantam microRNA in the larvae of transgenic and control flies. The studies were carried out using Real-time PCR adapted for microRNA (Chen et al., 2005; Kramer, 2011). The “yw” line with a normal bantam locus served as a positive control. We used the “Δban” line with the banΔ1 deletion as negative control. Larvae homozygous for the insertions were selected. Since individuals homozygous for the banΔ1 deletion die at the pupal stage, studies were carried out on larvae. As expected, in the “Δban” line, the mature microRNA bantam was not detected in larvae homozygous for banΔ1 (Fig. 4). The expression of bantam was reduced in both transgenic lines, “4.7” and “DREF”, compared to the control “yw”. A significant decrease in the expression level of mature microRNA in the “4.7” line seems surprising given that the viability of flies in the “4.7” line did not differ from the control. This may be explained by the fact that although in the “4.7” line, the level of bantam microRNA expression was significantly reduced, it remained at a sufficient level in all the tissues where it was necessary for fly survival. And in the “DREF” line, the bantam expression level was ubiquitously at a low, threshold level, which significantly affected viability. It is also possible that in the “DREF” line, bantam expression was reduced only in certain tissues critical for fly survival. The data obtained suggest that although the “DREF” transgene rescues the banΔ1 deletion, it does not contain all the regulatory elements required for full bantam expression.

The fundamental ability of the mutant transgene in the “DREF” line to “rescue” the banΔ1 deletion indirectly confirms that the introduced mutations did not destroy the binding site of the BEAF-32 protein. We have previously shown that destruction of the BEAF-32 protein binding site in the bantam regulatory region leads to death at the late pupal stage (Schwartz et al., 2019). 

Interestingly, in the “DREF” transgenic line, the viability of adult flies homozygous for banΔ1 was sex-dependent. The proportion of males homozygous for banΔ1 was only 30 % of all adult flies, which is significantly lower than in the “4.7” line and in the control ( р < 0.01). This may be explained by the fact that disruption of the DREF binding site in the bantam regulatory region has a greater effect on the viability of males than females. Another explanation could be the different levels of transgene activity on the X chromosome associated with dosage compensation. Despite the fact that males of the “DREF” line, homozygous for banΔ1, were less viable than females, they did not have problems with fertility.

Disruption of the DREF binding site in the bantam promoter region significantly reduces female fertility

**Fig. 5.**
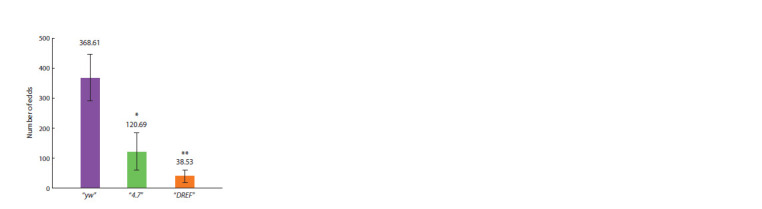
Fertility of transgenic fly line females. Average number of eggs per female from the control line “yw” and transgenic lines “4.7” and “DREF”, homozygous for the transgenes and for the banΔ1 deletion. ** р < 0.01; * р < 0.05.

We studied the fertility of females of transgenic lines, estimating the average number of eggs laid per female (see Materials and methods). In the “4.7” line, the fertility of females homozygous for banΔ1 was significantly reduced compared to females of the “yw” control line and amounted to 32.7 % of the fertility of control “yw” females, taken as 100 % (Fig. 5). The fertility of females homozygous for banΔ1 in the “DREF” line was only 10.5 %. These data indicate that the 4.7 fragment does not contain all the regulatory elements necessary for the normal progression of oogenesis, and the mutation of the binding site in the “DREF” line disrupts this process even more crucially

However, it should be taken into account that the life expectancy of females in transgenic lines was significantly reduced compared to control females of the “yw” line ( р < 0.05). Thus, in the “yw” line, 50 % of the females died on average on the 22nd day of the experiment, in the “4.7” line – on the 16th day, and in the “DREF” line – on the 10th day.

**Fig. 6.**
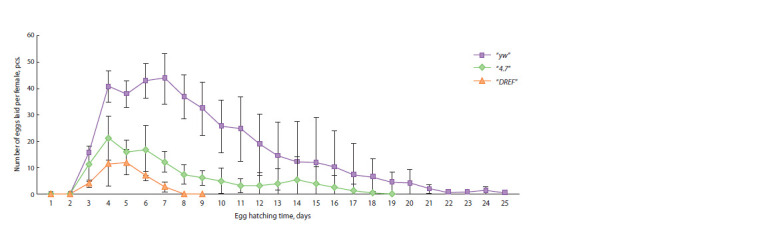
Temporal dynamics of female fertility. Average number of eggs laid per day per female in the control “yw” line and in the transgenic lines “4.7” and “DREF”, homozygous for transgenes and for the banΔ1 deletion

At the same time, a reduction in life expectancy was not the only explanation for the decrease in the number of eggs laid by females of transgenic lines. Analysis of the dynamics of egg laying showed that females of transgenic lines not only laid fewer eggs on each day of the experiment than females of the control “yw” line ( p < 0.001), but also finished laying eggs much earlier (Fig. 6). The reproductive period in females of the “4.7” line lasted on average 16.3 days ( p <0.01), and in “DREF” females – 8.3 days ( p < 0.001), while the reproductive period in control “yw” females was 24.7 days.

Disruption of the DREF binding site reduces bantam miRNA expression in adult fly ovaries

The early completion of egg laying in transgenic lines is similar to the previously described situation with inactivation of bantam microRNA in germline stem cells in the ovaries of adult flies (Shcherbata et al., 2007). According to the authors, in such females, about 14 % of germline stem cells left their niche per day. This can lead to both a general decrease in fertility and a shortening of the reproductive period.

**Fig. 7.**
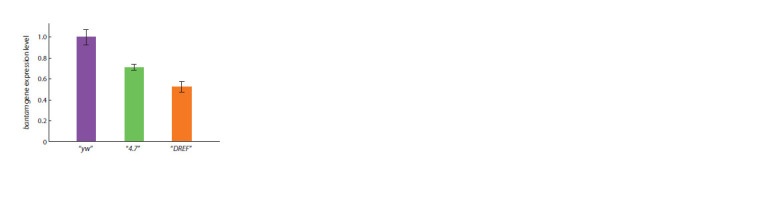
The expression of mature bantam microRNA in the ovaries of transgenic females The amount of mature microRNA bantam in the transgenic lines “4.7” and “DREF”, homozygous for the banΔ1 deletion. The expression of bantam in the control “yw” line was conditionally taken as one. The bantam expression levels were normalized to the expression level of the U6 snRNA reference gene.

We decided to test whether DREF binding to the bantam regulatory region actually affects the expression level of mature microRNA. Using qRT-PCR, we showed that the expression of bantam microRNA in the ovaries of females of the transgenic lines “4.7” and “DREF” was lower than in females of the control “yw” line (Fig. 7).

The results obtained are consistent with the fact that in both transgenic lines the fertility of females was reduced compared to control “yw” females. Moreover, in the “DREF” line, bantam expression was reduced not only compared to “yw”, but also compared to “4.7”. This indicates that DREF binding to the regulatory region of the bantam gene plays an important role in its expression in the ovaries, and it is the disruption of this binding that may explain the significant decrease in fertility in females of the “DREF” line.

## Conclusion

In this work, we tested the functionality of a potential binding site for the transcription factor DREF, found in the regulatory region of the bantam gene. Disruption of this DREF binding site has a significant impact on fly viability and female fertility. This is accompanied by a significant decrease in the expression of mature bantam microRNA in both whole larvae and the ovaries of adult flies. As was previously shown, DREF positively influences the activity of the Hippo cascade, thereby indirectly limiting the expression of bantam (Fujiwara et al., 2012; Vo et al., 2014). The effect of DREF binding to the promoter region of the bantam gene on its expression level suggests an additional level of complexity in the regulation of the expression of this microRNA.

A decrease in the number of eggs laid and a shortening of the reproductive period in females when the DREF binding site in the regulatory region of the bantam gene is disrupted suggests that DREF is also involved in the regulation of Drosophila oogenesis through bantam.

## Conflict of interest

The authors declare no conflict of interest.

## References

Andreenkov O.V., Andreenkova N.G., Volkova E.I., Georgiev P.G.,
Goncharova A.A., Pokholkova G.V., Demakov S.A. Ectopic tethering
of the Chromator protein in UAS>DBD(GAL4) system as approach
for studying of the insulator proteins in Drosophila melanogaster
polytene chromosomes. Tsitologiya = Cell and Tissue
Biology.
2016;58(6):493-497 (in Russian)

Brennecke J., Hipfner D.R., Stark A., Russell R.B., Cohen S.M.
bantam
encodes a developmentally regulated microRNA that controls
cell proliferation and regulates the proapoptotic gene hid in
Drosophila. Cell. 2003;113(1):25-36. DOI 10.1016/s0092-8674(03)
00231-9

Chen C., Ridzon D.A., Broomer A.J., Zhou Z., Lee D.H., Nguyen J.T.,
Barbisin M., Xu N.L., Mahuvakar V.R., Andersen M.R., Lao K.Q.,
Livak K.J., Guegler K.J. Real-time quantification of microRNAs
by stem-loop RT-PCR. Nucleic Acids Res. 2005;33(20):e179. DOI
10.1093/nar/gni178

Fujiwara S., Ida H., Yoshioka Y., Yoshida H., Yamaguchi M. The warts
gene as a novel target of the Drosophila DRE/DREF transcription
pathway. Am. J. Cancer Res. 2012;2(1):36-44

Herranz H., Pérez L., Martín F.A., Milán M. A Wingless and Notch
double-repression mechanism regulates G1-S transition in the Drosophila
wing. EMBO J. 2008;27(11):1633-1645. DOI 10.1038/
emboj.2008.84

Hipfner D.R., Weigmann K., Cohen S.M. The bantam gene regulates
Drosophila growth. Genetics. 2002;161(4):1527-1537. DOI
10.1093/genetics/161.4.1527

Huang J., Wu S., Barrera J., Matthews K., Pan D. The Hippo signaling
pathway coordinately regulates cell proliferation and apoptosis by
inactivating Yorkie, the Drosophila Homolog of YAP. Cell. 2005;
122(3):421-434. DOI 10.1016/j.cell.2005.06.007

Kolesnikova T.D., Posukh O.V., Andreyeva E.N., Bebyakina D.S.,
Ivankin
A.V., Zhimulev I.F. Drosophila SUUR protein associates
with PCNA and binds chromatin in a cell cycle-dependent manner.
Chromosoma. 2013;122(1-2):55-66. DOI 10.1007/s00412-012-
0390-9

Kramer M.F. Stem-loop RT-qPCR for miRNAs. Curr. Protoc. Mol.
Biol. 2011;95(1):15.10.1-15.10.15. DOI 10.1002/0471142727.mb
1510s95

Ku H.Y., Sun Y.H. Notch-dependent epithelial fold determines boundary
formation between developmental fields in the Drosophila antenna.
PLoS Genet. 2017;13(7):e1006898. DOI 10.1371/journal.
pgen.1006898

Nagata R., Akai N., Kondo S., Saito K., Ohsawa S., Igaki T. Yorkie
drives supercompetition by non-autonomous induction of autophagy
via bantam microRNA in Drosophila. Curr. Biol. 2022;32(5):
1064-1076.e4. DOI 10.1016/j.cub.2022.01.016

Oh H., Irvine K.D. Yorkie: the final destination of Hippo signaling.
Trends Cell Biol. 2010;20(7):410-417. DOI 10.1016/j.tcb.2010.04.
005

Ohler U., Liao G.C., Niemann H., Rubin G.M. Computational analysis
of core promoters in the Drosophila genome. Genome Biol. 2002;3:
research0087.1. DOI 10.1186/gb-2002-3-12-research0087

Peng H.W., Slattery M., Mann R.S. Transcription factor choice in the
Hippo signaling pathway: Homothorax and yorkie regulation of the
microRNA bantam in the progenitor domain of the Drosophila eye
imaginal disc. Genes Dev. 2009;23(19):2307-2319. DOI 10.1101/
gad.1820009

Qian J., Zhang Z., Liang J., Ge Q., Duan X., Ma F., Li F. The fulllength
transcripts and promoter analysis of intergenic microRNAs
in Drosophila melanogaster. Genomics. 2011;97(5):294-303. DOI
10.1016/j.ygeno.2011.02.004

Reddy B.V.V.G., Irvine K.D. Regulation of Drosophila glial cell proliferation
by Merlin–Hippo signaling. Development. 2011;138(23):
5201-5212. DOI 10.1242/dev.069385

Schwartz (Berkaeva) M.B., Pankova T.E., Demakov S.A. ADF1 and
BEAF-32 chromatin proteins affect nucleosome positioning and
DNA decompaction in 61C7/C8 interband region of Drosophila melanogaster
polytene chromosomes. Vavilov Journal of Genetics and
Breeding. 2019;23(2):154-159. DOI 10.18699/VJ19.475

Shcherbata H.R., Ward E.J., Fischer K.A., Yu J.Y., Reynolds S.H.,
Chen C.H., Xu P., Hay B.A., Ruohola-Baker H. Stage-specific differences
in the requirements for germline stem cell maintenance
in the Drosophila ovary. Cell Stem Cell. 2007;1(6):698-709. DOI
10.1016/j.stem.2007.11.007

Slattery M., Voutev R., Ma L., Nègre N., White K.P., Mann R.S. Divergent
transcriptional regulatory logic at the intersection of tissue
growth and developmental patterning. PLoS Genet. 2013;9(9):
e1003753. DOI 10.1371/journal.pgen.1003753

Vo N., Horii T., Yanai H., Yoshida H., Yamaguchi M. The Hippo pathway
as a target of the Drosophila DRE/DREF transcriptional regulatory
pathway. Sci. Rep. 2014;4:7196. DOI 10.1038/srep07196

Weng R., Cohen S.M. Control of Drosophila Type I and Type II central
brain neuroblast proliferation by bantam microRNA. Development.
2015;142(21):3713-3720. DOI 10.1242/dev.127209

Zhang X., Aksoy E., Girke T., Raikhel A.S., Karginov F.V. Transcriptome-
wide microRNA and target dynamics in the fat body during the
gonadotrophic cycle of Aedes aegypti. Proc. Natl. Acad. Sci. USA.
2017;114(10):E1895-E1903. DOI 10.1073/pnas.1701474114

